# Primary melanoma of the small bowel revealed by gastrointestinal bleeding: a case report

**DOI:** 10.1186/s13256-016-1119-9

**Published:** 2016-12-01

**Authors:** B. Ait Idir, A. Riany, A. Jahid, B. Chad

**Affiliations:** 1 Surgery Unit B, Ibn Sina University Hospital, Rabat, Morocco; 2 Pathology Unit, Ibn Sina University Hospital, Rabat, Morocco

**Keywords:** Intestinal melanoma, Primary melanoma of the small bowel, Gastrointestinal bleeding

## Abstract

**Background:**

Primary melanoma of the small bowel is extremely rare. Only a limited number of cases have been described in the literature. Mostly, the small intestine is affected by metastatic tumors of other primary lesions, especially cutaneous.

**Case presentation:**

We report the case of a 75-year-old North African woman with a small bowel melanoma. The diagnosis was made by histological examination and immunohistochemical profile matching after a segmental small bowel resection. Postoperative investigations looking for cutaneous, gastrointestinal or ocular primary lesions found no abnormalities.

**Conclusions:**

The diagnosis of primary small bowel melanoma can be retained although it remains difficult to exclude the possibility of metastatic melanoma.

## Background

Melanoma is a malignant tumor developed from melanocytes which are usually located in the skin, the eye’s choroid, the meninges, and the anal margin. Melanoma of the gastrointestinal tract represent 1 to 3% of the digestive cancers [1, 2], it is essentially a metastasis of a cutaneous, ocular, or anal primary lesion [3]. Primary melanoma of the small bowel is exceptional, only few cases have been reported in the literature due to the difficulty in excluding another primary cancer [2, 4]. Regardless of its primary or secondary character, intestinal melanoma remains more aggressive with a poor prognosis compared to other nondigestive locations. The median overall survival is 4 to 6 months with a survival rate of less than 10% at 5 years [3, 5].

With regard to therapeutic approach, there is no real consensus in the curative treatment of primary melanoma of the gastrointestinal tract. Surgery remains an essential approach for this disease, since effective adjuvant systemic therapies are without benefit for overall survival [6]. The optimal surgical technique consists of a carcinologic resection of the tumor.

## Case presentation

**Fig. 1:**
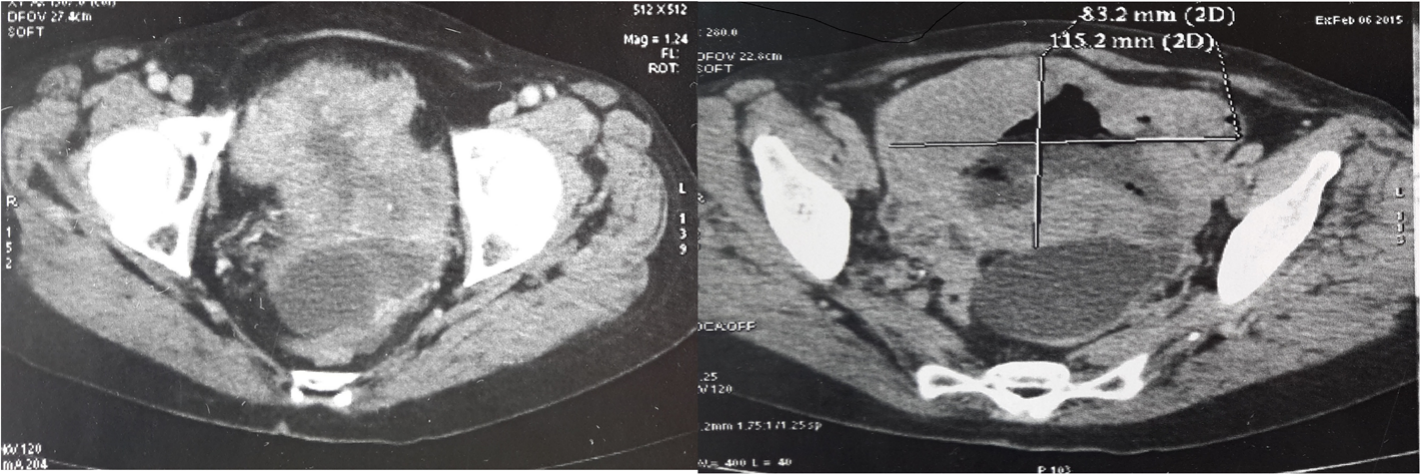
Computed tomography scan showing a pelvic mass measuring11 × 9 cm involving the distal small bowel loops, the bladder dome, and the uterine body without peritoneal effusion

**Fig. 2:**
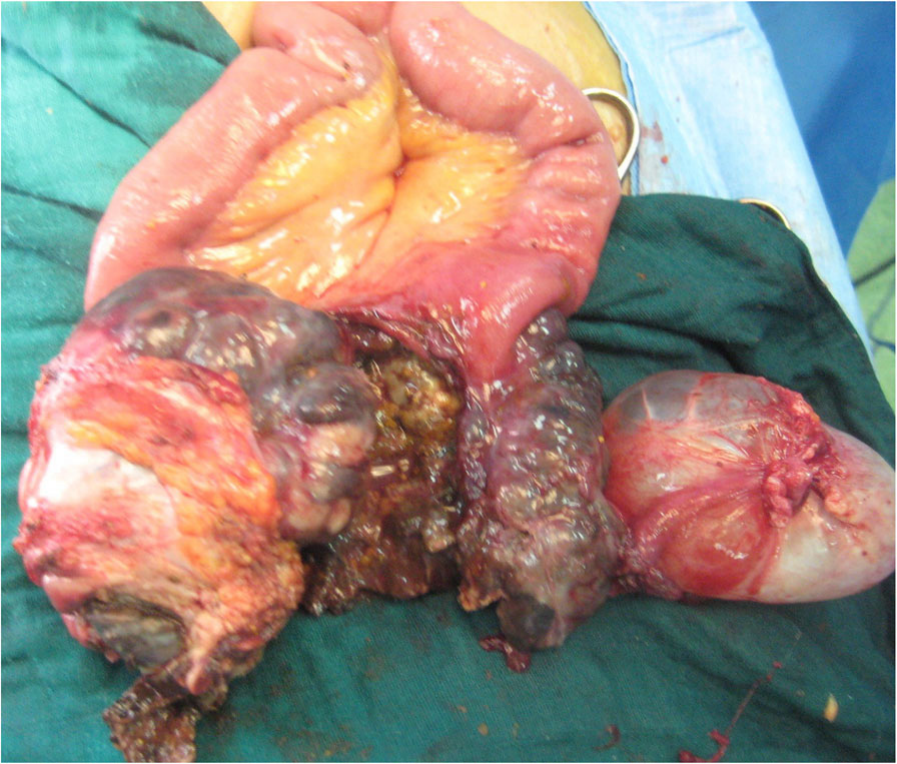
Intraoperative finding revealing a pseudoaneurysmal tumor of the small intestine involving the left ovary

**Fig. 3:**
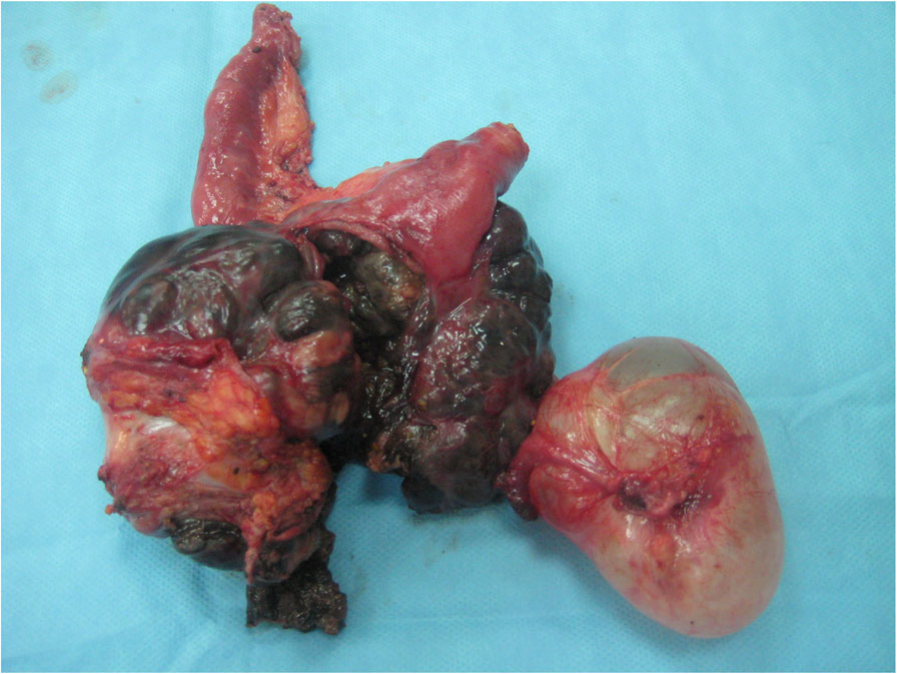
Limited small bowel resection extended to the left annexes and to a portion of the bladder

**Fig. 4:**
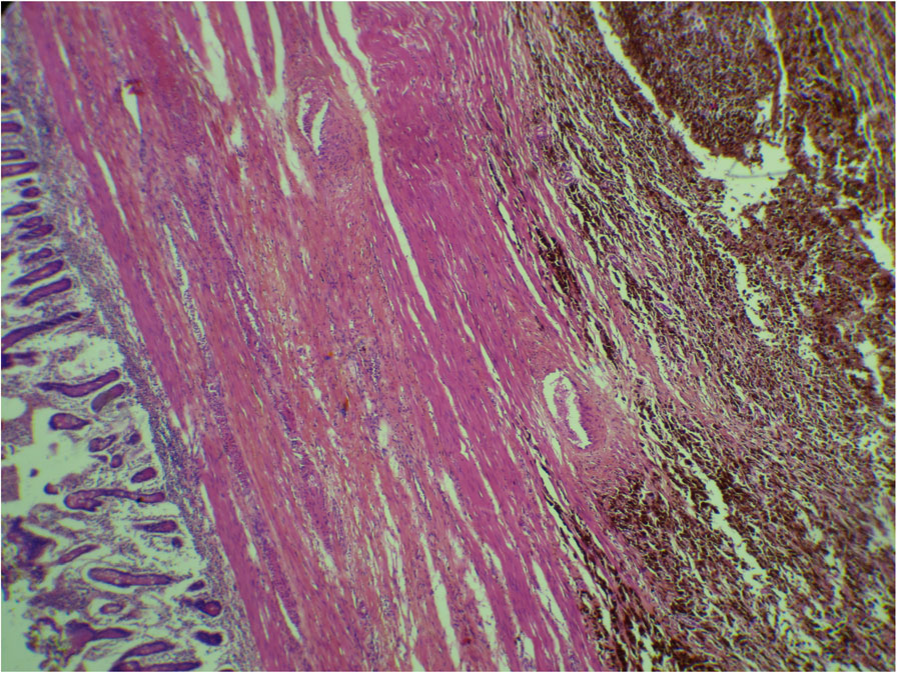
Large tumoral malignant cell proliferation with melanin pigmentations infiltrating the small bowel wall. Hematoxylin and eosin, ×100

**Fig. 5:**
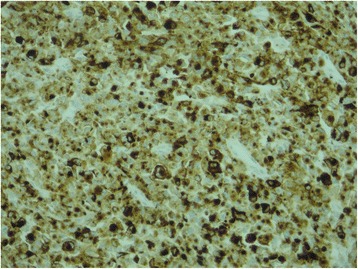
Tumor cells are positive in immunohistochemical staining for HMB-45

**Fig. 6:**
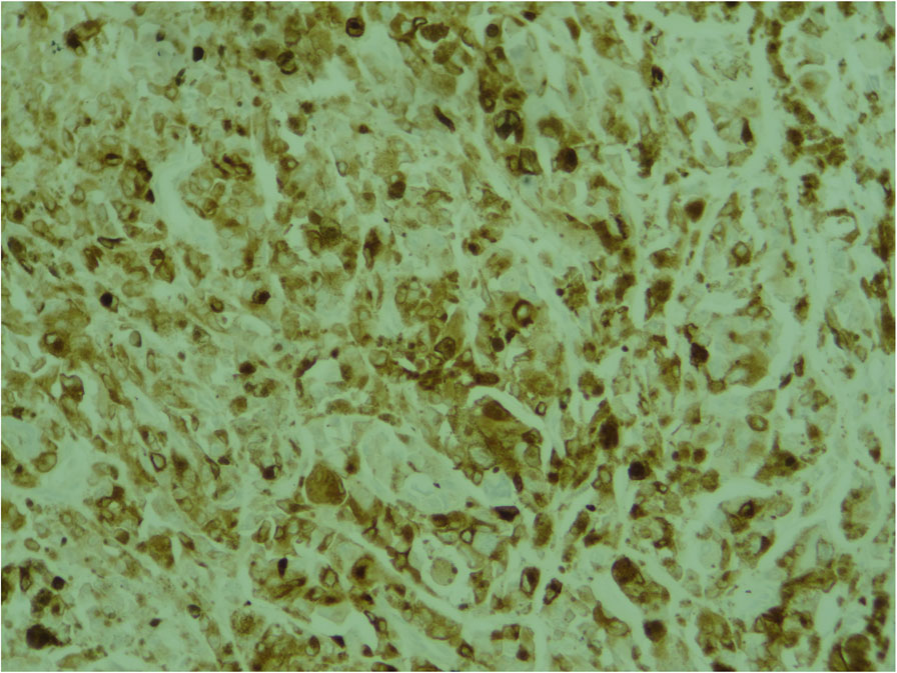
Tumor cells are positive in immunohistochemical staining for S-100

We report the case of a 75-year-old North African woman with no notable medical history, admitted for pelvic pain lasting for the last 4 months and unrelieved by analgesics; the transit was undisturbed. The symptoms were aggravated by melena and asthenia that motivated the medical consultation. A clinical examination at admission found a patient with a stable hemodynamic status, an abdominal examination revealed a palpable and mobile pelvic mass. Laboratory tests showed a hypochromic and microcytic anemia with low hemoglobin and hematocrit levels rating at 7.3 g/dL and 21%. An abdominal computed tomography (CT) scan showed a large pelvic mass measuring 11 × 9 cm involving the distal small bowel loops, the bladder dome, and the uterine body without peritoneal effusion (Fig. 1). After primary care, our patient underwent an exploratory laparotomy. The intraoperative finding was a brownish pseudoaneurysmal mass of the small bowel located 80 cm from the Treitz’s angle; this mass was invading the bladder dome and the left ovary and closely adhering to the uterus (Fig. 2). Limited small bowel resection with 10 cm margins on both sides of the tumor extended to the left annexes and to a portion of the bladder with end-to-end anastomosis was achieved. The postoperative management was uneventful and our patient was discharged on day 6. The surgical specimen was 18 cm in length and included a black solid tumor with exophytic growth infiltrating the small bowel wall until the mucosa (Fig. 3). A histological examination revealed a malignant proliferation of large cells with prominent round nuclei and a cytoplasm with eosinophilic spots or the seat of melanin pigments. Tumor necrosis was estimated at 30%, the rest of the ileum was the seat of chronic ileitis (Fig. 4). The immunohistochemical profile showed an intense and diffuse cytoplasmic positivity for HMB-45 antigens and for PS-100, suggesting a malignant melanoma (Figs. 5 and 6). An etiological investigation in search of a primary tumor of the small bowel melanoma was negative, an anoscopy, examinations of eyes and skin with multiple cutaneous biopsies were performed without finding any melanoma lesion.

## Discussion

Melanoma develops in the melanocytes, which are dendritic cells present in the skin, the eye, and the epithelium of the nasal cavity, oropharynx, anus, vagina, and urinary tract. In addition to these sites, it was proven by immunoperoxidase studies that melanocytes are present in Meckel’s diverticulum [7, 8]. Normally, the small intestine and the colon do not contain melanocytes. Embryologically, they arise from neural crest melanoblasts which migrate to the distal ileum through the umbilical-mesenteric canal [7, 9]. They differentiate by amine precursor uptake and decarboxylation (APUD), and can undergo neoplastic transformation in noncutaneous sites [10]. According to this theory, the ileum is the most common site for the development of primary melanoma of the small intestine, although some authors still deny the existence of primary melanoma in the gastrointestinal tract. They argue that primary cutaneous tumors can regress before metastatic manifestations or they are too small to be identified by clinical and laboratory examinations [2]. Primary or secondary gastrointestinal melanoma is difficult to establish resulting in many controversies [11].The gastrointestinal tract is the most common site of cutaneous melanoma metastasis [4, 10]. Usually asymptomatic, metastases are diagnosed at autopsy in 58% of patients with cutaneous melanoma [9], they may be clinically detected only after treatment of primary melanoma, or spontaneous regression [5], affecting primarily the small intestine, stomach, and colon [4, 12]. However, some gastrointestinal melanomas remain undocumented and without evidence of a primary lesion, cutaneous or elsewhere, even after a thorough examination [13].The incidence of metastatic gastrointestinal melanoma of unknown primary origin is from 4 to 9% in case series [14]. In order to distinguish between primary and metastatic intestinal melanoma, Bender et al. were able to identify four different types of metastatic melanoma of the small intestine based solely on histopathological features: cavitary, infiltrating, eccentric, and polypoid [15]. Lymphocytic infiltrate with melanophages, restorative fibrosis, and a vascular proliferation present in the dermis are commonly seen in intestinal metastatic melanoma developed after spontaneous regression of the primary cutaneous lesion [6]. To make the diagnosis of primary malignant melanoma of the small intestine, other authors recommend excluding any history of melanoma in the other major sites. Sacks et al. established three diagnostic criteria: (1) single lesion, (2) no metastatic location other than in the regional lymph nodes, (3) relapse-free survival of more than 1 year after diagnosis [16].

In our case, histological study confirmed the diagnosis of intestinal melanoma with concordant immunohistochemical profile; our patient had no history of cutaneous melanoma, and etiological examination found no other melanoma lesion. At 1-year follow-up, clinical examination and abdominal CT scan control showed no intraperitoneal signs of recurrence. The diagnosis of a primary malignant melanoma of the small bowel has been established.

Adjuvant systemic therapy has a limited role, different chemotherapy regimens have been applied but the response rates were extremely low [6]. In our case, no adjuvant therapy was administered.

## Conclusions

The diagnosis of primary intestinal melanoma can be confirmed based on the criteria described in the literature; however, early diagnosis and surgical resection are essential for improving the prognosis.
